# EIGHT-YEAR CHANGES IN DEFICIT ACCUMULATION FRAILTY: RELATION TO GLYCEMIC CONTROL AND DIABETES MEDICATION USE

**DOI:** 10.1093/geroni/igac059.853

**Published:** 2022-12-20

**Authors:** Felicia Simpson, Edward Boyko, Chloe Ferris, Jamie Justice, Stephen Kritchevsky, Jeanne Mccaffery, Scott Pilla, Mark Espeland

**Affiliations:** Winston-Salem State University, Winston-Salem, North Carolina, United States; University of Washington School of Medicine, Seattle, Washington, United States; Washington University School of Medicine, St. Louis, Missouri, United States; Wake Forest School of Medicine, Winston-Salem, North Carolina, United States; Wake Forest School of Medicine, Winston-Salem, North Carolina, United States; University of Connecticut, Storrs, Connecticut, United States; The Johns Hopkins University School of Medicine, Baltimore, Maryland, United States; Wake Forest School of Medicine, Winston-Salem, North Carolina, United States

## Abstract

Type 2 diabetes mellitus (T2DM) has been linked to accelerated biological aging and the accumulation of health deficits. It is unknown whether glycemic control can slow the progression of aging, as expressed by deficit accumulation frailty indices (FIs). We examined the cross-sectional and longitudinal associations that glycemic control, diabetes medication use, and weight change (predictors) had with a FI calculated as the percent of 36 deficits in behavioral, functional, and clinical characteristics (outcome). We drew data from 4177 participants across 8 years of follow-up in the Look AHEAD clinical trial of a multidomain intensive lifestyle intervention in individuals aged 45-76 years with T2DM and overweight or obesity. At baseline, the means(SD) FI(as a percent) for participants grouped as HbA1c< 7%, 7-7.9%, and >8% were: 19.93(6.45), 20.82(6.89), and 21.53(7.27), p< 0.001. Compared with HbA1c>8%, HbA1c< 7% at baseline was associated with 23% less mean 8-year FI progression: 2.42(7.35) vs 3.14(8.02), p< 0.001). Maintaining average HbA1c< 7% vs >8% during follow-up was associated with 47% less 8-year FI progression: 2.05(7.35) vs 3.14(8.02), p< 0.001. With adjustment for HbA1c, sustained weight loss >5% compared with weight gain >5% was associated with an 81% reduction in 8-year FI progression: 1.18(7.25) vs 3.89(8.09), p< 0.001. Use of metformin across >50% of annual visits was associated with a 30% reduction in 8-year progression of FI as compared with less use or no use: 2.25(7.27) vs 3.22(7.90), p=0.002. We conclude that better control of HbA1c, and sustained weight loss (>5%) and metformin use, may slow the accelerated aging associated with T2DM.

